# Supplementary material for: Identification of photoperiod-induced specific miRNAs in the adrenal glands of Sunite sheep (Ovis aries)
Source: Front Vet Sci. 2022 Jul 22;9:888207. doi: 10.3389/fvets.2022.888207 (PMC9354845; doi:10.3389/fvets.2022.888207)
Supplement: Supplementary file 1 [file Data_Sheet_1.ZIP › Supplementary file/miRDeep2 file.zip!/miRDeep2 file/Novel_Structure/LP42A3/Novel_200.pdf]

Provisional ID	:	NC_019459.2_2506
Score total	:	2.6
Score for star read(s)	:	-1.3
Score for read counts	:	0
Score for mfe	:	2.3
Score for randfold	:	1.6
Score for cons. seed	:	
Total read count	:	78
Mature read count	:	78
Loop read count	:	0
Star read count	:	0

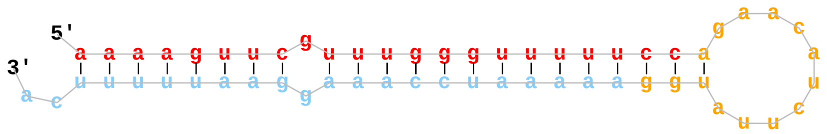

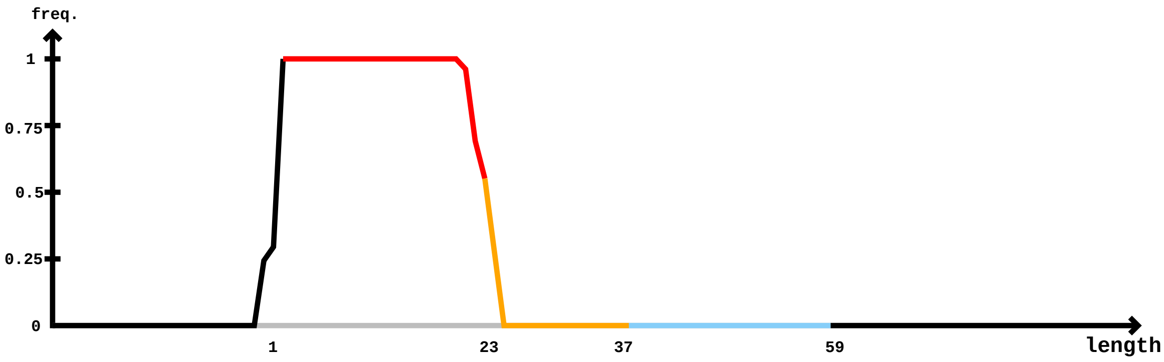

Mature **Star**

```
5'- ucaaauaauauuggguuggcugaaaaguucguuuggguuuuuccagaacaucuuauggaaaaauccaaaggaauuuuucagccaacacaauauuaacuuucacagcuuugu   -3'    exp
    .....((((((((.((((((((((((((((.((((((((((((((..........)))))))))))))).)))))))))))))))).))))))))................   reads    mm    sample
    ....................ugaaaaguucguuuggguuuu......................................................................   1        0     seq
    ....................ugaaaaguucguuuggguuuuu.....................................................................   18       0     seq
    .....................gaaaaguucguuuggguuuuu.....................................................................   3        0     seq
    .....................gaaaaguucguuuggguuuuuc....................................................................   1        0     seq
    ......................aaaaguucguuuggguuuC......................................................................   2        1     seq
    ......................aaaaguucguuuggguuuuuU....................................................................   10       1     seq
    ......................aaaaguucguuuggguuuuucG...................................................................   2        1     seq
    ......................aaaaguucguuuggguuuuucA...................................................................   41       1     seq
```
